# S207. TDCS AS FUTURE TREATMENT OPTION FOR SCHIZOPHRENIA PATIENTS - A NEUROPHYSIOLOGICAL INVESTIGATION OF INDUCED PLASTICITY OVER MOTOR AND PREFRONTAL CORTEX USING SLORETA

**DOI:** 10.1093/schbul/sby018.994

**Published:** 2018-04-01

**Authors:** Nina Filipova, Alkomiet Hasan, Daniel Keeser, Wolfgang Strube

**Affiliations:** Ludwig-Maximilians-University of Munich

## Abstract

**Background:**

Transcranial direct current stimulation (tDCS) is a non-invasive, plasticity-inducing brain stimulation technique that can induce long-lasting excitability changes in the motor cortex and has been discussed as an alternative treatment option for patients with schizophrenia. Therefore, the aim of the present study was to detect electrophysiological correlates after motor cortical and prefrontal tDCS in order to improve the understanding of tDCS-mechanisms. Anodal and cathodal tDCS was applied over motor cortex (M1) and dorsolateral prefrontal cortex (DLPFC), which is known as one major region of interest considering neurobiology of psychosis. Thus, we looked for tDCS-induced source-localized activity changes in resting EEG by using sLORETA (standardized low-resolution brain electromagnetic tomography) and compared the effects of motor and prefrontal cortex.

**Methods:**

A total of 20 healthy volunteers were examined within five sessions (within-subject design). Anodal tDCS (1mA, 13 minutes) and cathodal tDCS (1mA, 9 min) were applied over M1 and respectively DLPFC. In addition, there was a sham tDCS of DLPFC. Transcranial magnetic stimulation (TMS) was performed before and after motor cortical tDCS in order to generate motor evoked potentials (MEP) as periphery indicators of motor cortical plasticity. A 6-minute resting EEG was performed before and after each tDCS treatment. EEG data was then investigated by sLORETA for source-localized brain activity changes.

**Results:**

After tDCS over M1, the expected increase of MEP amplitude after anodal tDCS and reduction after cathodal tDCS could be measured. Following anodal tDCS over M1 an increased activity was found in the area of precuneus in EEG frequency band alpha. After cathodal tDCS over M1 an activity decrease was seen in frequency band alpha, beta and total power, which could be localized in insula and temporal gyrus.

After anodal as well as cathodal tDCS over DLPFC decreased activities could be measured in most frequency bands (e.g. delta, theta, alpha, beta, total power). Most of these changes were found in frontal lobe, anterior cingulate or insula. Unexpectedly there were also significant changes after sham tDCS in all frequency bands. However, these were measured mostly in right-sided temporal lobe, which could be due to jaw muscle artefacts.

**Discussion:**

The polarity-specific tDCS effects, which can be demonstrated in motor cortex, cannot be seen in prefrontal cortex; instead we detected a polarity-independent frontal modulation. This lack of prefrontal polarity specificity may be explained by a more complex mode of action in frontal cortex. This is consistent with the variable results of prefrontal tDCS in other publications. As the effects from motor cortical studies cannot easily be transferred to the frontal system in healthy subjects, one could speculate that in schizophrenic patients the responses to prefrontal tDCS might be even more difficult to predict. Further investigations are required to evaluate the heterogeneity of source-localized tDCS-effects and to understand prefrontal mechanisms, so that frontal tDCS may be used as a future treatment in schizophrenia patients.

